# Secondary hyperparathyroidism in patients on dialysis therapy in 2025

**DOI:** 10.1590/2175-8239-JBN-2025-E006en

**Published:** 2025-04-25

**Authors:** Tilman B. Drüeke

**Affiliations:** 1 Institut National de la Santé et de la Recherche Médicale (Inserm), Centre de Recherche en Epidémiologie et Santé des Populations (CESP), Villejuif, France.; 2 Paris-Saclay University (UPS), Gifsur-Yvette, France.; 3 Versailles Saint-Quentin-en-Yvelines University (UVSQ), Versailles, France.

**Figure 1:**
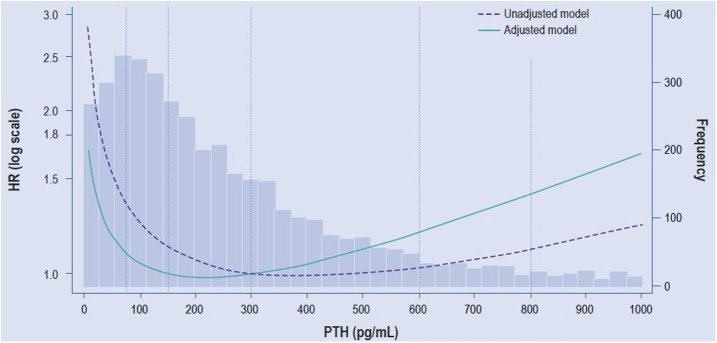
Relative risk of all-cause mortality for iPTH comparing baseline versus time-dependent Cox regression using fractional polynomials.

Chronic kidney disease (CKD) is a risk factor for several complications. The most prominent of these are arterial hypertension, cardiovascular and cerebrovascular disease, anemia, and a variety of pathologies secondary to metabolic and endocrine disturbances. The latter comprise the CKD-associated mineral and bone disorder (CKD-MBD). This term was created in 2009 by a Kidney Disease: Improving Global Outcomes (KDIGO) Work Group to reflect a broad clinical syndrome encompassing mineral, bone, and calcific cardiovascular abnormalities that develop as a complication of CKD^1^. In a very recent report, the KDIGO members of a controversy conference stated that the term CKD-MBD may no longer best reflect currently available evidence related to diagnosis and treatment of this patient population and proposed that future guideline efforts should instead consider mineral homeostasis and deranged endocrine systems within a context of 2 clinical syndromes: CKD-associated osteoporosis, encompassing increased fracture risk in patients with CKD; and CKD-associated cardiovascular disease^2^. Regardless new future definitions, secondary hyperparathyroidism (SHPT) is the main driver of high-turnover bone disease in CKD-MBD and its sometimes dramatic consequences. It further contributes to cardiovascular disease and several other CKD-associated complications, enhances the risk of mortality (Figure 1)^3^, and contributes to the progression of CKD in a vicious circle^4^.

Abbreviations - HR: hazard ratio. iPTH: intact parathyroid hormone.

Notes - Values <0.5% percentile and >99.5% percentile were removed from both models (baseline model = 29 observations removed and time-dependent model = 838 observations removed). iPTH values >1000 pg/mL not shown. Baseline model: log (HR) = −0.28iPTH0.5 + 0.04iPTH0.5 log iPTH + βkÅ~k (P = 0.001); time-dependent model: log (HR) = −0.38 log iPTH + 0.05iPTH0.5 + βkÅ~k (P < 0.001). Frequency: number of patients for each serum iPTH level.

Source - Floege J et al, modified (ref. 3).

Serum parathyroid hormone (PTH) increases with the progression of CKD. The velocity of the increase is highly variable from patient to patient, depending on the type of nephropathy and concomitant changes in closely intertwined factors such as calcium, phosphate, 1,25-dihydroxy vitamin D, fibroblast growth factor-23, alpha-klotho, wnt-inhibitors, and uremic toxins^5^. A persistent problem is the precise definition of SHPT of CKD. The optimal serum PTH levels for patients with CKD stages G3-G5D are unknown. Theoretically, PTH should progressively increase from one CKD stage to another, but things are complicated by the fact that the effects of PTH on tissues such as bone and the cardiovascular system depend not only on the circulating level but also on the tissue response to its action. As far as bone is concern, CKD leads to a relative skeletal resistance to the action of PTH, and progressively increasing PTH levels are necessary to overcome this resistance^6^. In practice, this means that patients with CKD require slightly to moderately elevated PTH levels rather than normal levels to achieve an appropriate tissue response.

In this issue of the journal, Pelepenko et al.^7^ report the results of a nationwide survey, the aim of which was to update a 2011 census on the prevalence of SHPT among Brazilian patients on dialysis and to evaluate medical and surgical treatment access for this complication. The authors should be congratulated for the effort made to obtain nationwide information on this topic, considering the difficulties encountered with this type of investigation in a geographically widespread country with a large number of dialysis centers and patients.

Reading the title of the manuscript, one would expect a report on the entire range of serum PTH values. However, the authors chose to restrict their survey to those patients who had PTH levels either above 600 pg/mL or below 100 pg/mL. This was a wise decision given the KDIGO definition of a grey zone of PTH target levels 2–9 times the upper limit of normal^1^, corresponding roughly to the 100 and 600 pg/mL exclusion criteria used in the survey. The absence of precise information on PTH measurement assays and normal values in the different dialysis centers must also have played a role in this decision.

The observation that among the 23,535 patients of the survey, the prevalence of high PTH levels (>600 pg/mL) was 19.7% and that of extremely high PTH levels (>1,000 pg/mL) was 8.9% is interesting. How this compares to previous reports from other countries on the prevalence of SHPT in general and of severe SHPT in particular is almost impossible to say. This greatly depends on the definition of the diagnostic thresholds used for PTH, which has no commonly accepted standards, and on available treatment modalities. One possible approach to the dilemma is to examine the prevalence of surgical parathyroidectomy, the definitive therapy for uncontrolled SHPT, provided that this procedure is commonly available. A recent report from Japan, a country with no restrictions on parathyroid surgery, showed a 10.4% prevalence of parathyroidectomy between January 2008 and December 2009 among 894 patients on hemodialysis with a median intact PTH level of 588 pg/mL, compared with an 89.6% prevalence of cinacalcet therapy among 2,682 patients on hemodialysis with a median intact PTH level of 566 pg/mL. Interestingly, parathyroidectomy was associated with a lower risk of mortality compared with cinacalcet^8^.

In the survey by Pelepenko et al.^7^, only 2.7% of the patients on hemodialysis in a comparable range of high circulating PTH levels underwent parathyroidectomy. As stated by the authors, this low rate is worrisome, especially when considering limited accessibility to efficacious medications for SHPT management due to high cost, which greatly increases the risk of serious complications. We have shown previously that cinacalcet treatment in patients on hemodialysis with a median intact plasma PTH of 690 pg/mL allowed halving the need for parathyroidectomy compared with placebo, from 14% to 7%^9^.

Regarding the indications for parathyroidectomy, not everyone would agree with the authors’ statement in the Discussion that patients on dialysis with PTH levels >1,000 pg/mL require parathyroid surgery. In many of these patients, medical treatment may allow levels to be lowered into the “grey zone”, i.e. <600 pg/mL. In our opinion, surgical parathyroidectomy is only indicated in patients for whom medical treatment is unsuccessful^5^.

Pelepenko et al.^7^ rightly mention several limitations of their survey. A first important limitation is that only 13% of the dialysis facilities in Brazil returned the survey questionnaire, with almost half of them located in the southeast region of the country. Thus the findings may not reflect the northern regions with a lower economic status. Second, the survey does not provide insight into the causes and factors involved in the difficulties related to access to clinical and surgical treatment for SHPT. Third, no information is given on the impact of inadequate control of SHPT on patient outcomes. Fourth, the insufficient availability of head and neck surgeons is somewhat surprising since this is probably the cheapest treatment option for severe SHPT. Other limitations include the lack of precise information on dialysis therapy modalities other than hemodialysis, and the lack of discussion on to the 18.7% of patients with inappropriately normal or low PTH levels (<100 pg/mL) in whom mortality risk is increased as well^4^.

Notwithstanding these limitations, the survey by Pelepenko et al.^7^ is a useful document on the present condition regarding SPTH in CKD and its management in Brazil. It will hopefully encourage governmental agencies to make appropriate healthcare decisions aimed at further improving patient outcomes.
