# Supplementary material for: Secondary hyperparathyroidism in patients on dialysis therapy in 2025
Source: J Bras Nefrol. 2025 Apr 25;47(2):e2025E006. doi: 10.1590/2175-8239-JBN-2025-E006en (PMC12043330; doi:10.1590/2175-8239-JBN-2025-E006en)
Supplement: Translation

# Translation: Hiperparatireoidismo secundário em pacientes submetidos à terapia de diálise em 2025

**DOI:** 10.1590/2175-8239-JBN-2025-E006pt

**Authors:** Tilman B. Drüeke

**Affiliations:** 1 Institut National de la Santé et de la Recherche Médicale (Inserm), Centre de Recherche en Epidémiologie et Santé des Populations (CESP), Villejuif, France.; 2 Paris-Saclay University (UPS), Gifsur-Yvette, France.; 3 Versailles Saint-Quentin-en-Yvelines University (UVSQ), Versailles, France.

A doença renal crônica (DRC) é um fator de risco para diversas complicações. Entre elas, as mais proeminentes são a hipertensão arterial, as doenças cardiovasculares e cerebrovasculares, a anemia e uma variedade de patologias secundárias a distúrbios metabólicos e endócrinos. Essas últimas compreendem o distúrbio mineral e ósseo associado à DRC (DMO-DRC), termo introduzido em 2009, por um grupo de trabalho do KDIGO (*Kidney Disease: Improving Global Outcomes*), para refletir uma ampla síndrome clínica que abrange anormalidades cardiovasculares minerais, ósseas e de calcificação que se desenvolvem como uma complicação da DRC^1^. Em um relato bastante recente, os membros de uma Conferência de Controvérsias do KDIGO afirmaram que o termo DMO-DRC pode não mais refletir adequadamente as evidências atualmente disponíveis relacionadas ao diagnóstico e ao tratamento dessa população de pacientes. Como alternativa, propuseram que os esforços de futuras diretrizes considerem a homeostase mineral e os sistemas endócrinos alterados dentro do contexto de duas síndromes clínicas: osteoporose associada à DRC, que abrange o risco aumentado de fraturas em pacientes com DRC; e doença cardiovascular associada à DRC^2^. Independentemente das futuras novas definições, o hiperparatireoidismo secundário (HPTS) é o principal causador da doença óssea de alto *turnover* na DMO-DRC e de suas, por vezes, graves consequências. Além disso, contribui para a doença cardiovascular e diversas outras complicações associadas à DRC, aumenta o risco de mortalidade (Figura 1)^3^ e, em um círculo vicioso, contribui para a progressão da DRC^4^.

**Figura 1:** Risco relativo de mortalidade por todas as causas para iPTH comparando regressão de Cox basal versus tempo-dependente, utilizando polinômios fracionários.

Abreviações - HR: razão de risco. iPTH: paratormônio intacto.

Notas - Valores inferiores ao percentil 0,5% e superiores ao percentil 99,5% foram removidos de ambos os modelos (modelo basal = 29 observações removidas e modelo dependente do tempo = 838 observações removidas). Valores de iPTH >1000 pg/mL não mostrados. Modelo basal: log (HR) = −0,28iPTH0,5 + 0,04iPTH0,5 log iPTH + βkÅ~k (P = 0,001); modelo dependente do tempo: log (HR) = −0,38 log iPTH + 0,05iPTH0,5 + βkÅ~k (P < 0,001). Frequência: número de pacientes para cada nível sérico de iPTH.

Fonte - Floege J et al, modificado (ref. 3).

O paratormônio (PTH) sérico aumenta à medida que a DRC progride. A velocidade do aumento apresenta grande variação de um paciente para outro, dependendo do tipo de nefropatia e das alterações concomitantes de fatores estreitamente interligados, como cálcio, fosfato, 1,25-dihidroxi vitamina D, fator de crescimento de fibroblastos-23, α-klotho, inibidores de Wnt e toxinas urêmicas^5^. Uma questão persistente e problemática é a definição precisa de HPTS na DRC. Os níveis séricos ideais de PTH para pacientes nos estágios G3-G5D da DRC ainda não são conhecidos. Teoricamente, o PTH deveria aumentar progressivamente de um estágio de DRC para outro, mas a situação é complicada devido ao fato de que os efeitos do PTH em tecidos como os ossos e o sistema cardiovascular dependem não apenas do nível circulante, mas também da resposta do tecido à sua ação. No que se refere ao osso, a DRC induz uma resistência óssea relativa à ação do PTH, exigindo níveis progressivamente mais elevados desse hormônio para superar essa resistência^6^. Na prática, isso significa que, em pacientes com DRC, níveis de PTH não normais, mas de leve a moderadamente elevados, são necessários para obter uma resposta tecidual adequada.

Nesta edição da revista, Pelepenko et al.^7^ relatam os resultados de uma pesquisa nacional, cujo objetivo foi atualizar um censo anterior, realizado em 2011, sobre a prevalência de HPTS entre pacientes brasileiros em diálise e avaliar o acesso ao tratamento médico e cirúrgico para essa complicação. Os autores merecem ser parabenizados pelo esforço empreendido para obter informações de âmbito nacional sobre esse tema, considerando as dificuldades inerentes a esse tipo de investigação em um país geograficamente vasto, com muitos centros de diálise e pacientes.

Ao ler o título do manuscrito, espera-se um relato sobre todo o intervalo de valores de PTH. No entanto, os autores optaram por restringir sua pesquisa somente àqueles pacientes com níveis de PTH acima de 600 pg/mL ou abaixo de 100 pg/mL. Essa foi uma decisão sensata, dada a definição do KDIGO de uma zona cinzenta de níveis-alvo de PTH de 2 a 9 vezes o limite superior do normal^1^, o que corresponde aproximadamente aos critérios de exclusão de 100 e 600 pg/mL utilizados na pesquisa. A ausência de informações precisas sobre ensaios de medição de PTH e valores normais nos diferentes centros de diálise provavelmente também influenciou essa decisão.

É interessante observar que, entre os 23.535 pacientes da pesquisa, a prevalência de níveis elevados de PTH (>600 pg/mL) foi de 19,7%, e a de níveis extremamente elevados de PTH (>1.000 pg/mL) foi de 8,9%. É quase impossível dizer como isso se compara a relatos anteriores de outros países sobre a prevalência de HPTS em geral e de HPTS grave em particular. Isso depende em grande parte da definição dos limites diagnósticos utilizados para o PTH, sem padrões comumente aceitos, e das modalidades de tratamento disponíveis. Uma possível abordagem para lidar com esse dilema é examinar a prevalência da paratireoidectomia cirúrgica, a terapia definitiva para o HPTS não controlado, desde que esse procedimento esteja normalmente disponível. Um recente relato do Japão, um país sem limitações para a cirurgia de paratireoide, mostrou uma prevalência de 10,4% de paratireoidectomia entre janeiro de 2008 e dezembro de 2009, entre 894 pacientes em hemodiálise, com um nível mediano de PTH intacto de 588 pg/mL, em comparação com uma prevalência de 89,6% de terapia com cinacalcete, entre 2.682 pacientes em hemodiálise, com um nível mediano de PTH intacto de 566 pg/mL. É interessante notar que a paratireoidectomia, quando comparada ao cinacalcete, foi associada a um menor risco de mortalidade^8^.

Na pesquisa de Pelepenko et al.^7^, apenas 2,7% dos pacientes em hemodiálise, em um intervalo comparável de níveis elevados de PTH circulante, foram submetidos à paratireoidectomia. Como afirmado pelos autores, essa baixa taxa é preocupante, especialmente quando se considera o acesso limitado a medicamentos eficazes para o manejo do HPTS devido ao alto custo, o que aumenta consideravelmente o risco de complicações graves. Mostramos anteriormente que o tratamento com cinacalcete em pacientes em hemodiálise, com um PTH intacto plasmático mediano de 690 pg/mL, permitiu reduzir pela metade a necessidade de paratireoidectomia em comparação com o placebo, passando de 14% para 7%^9^.

Quanto às indicações para a paratireoidectomia, nem todos concordariam com a afirmação dos autores na seção **Discussão** de que pacientes em diálise com níveis de PTH >1.000 pg/mL necessitam de cirurgia de paratireoide. Em muitos desses pacientes, o tratamento médico pode permitir a redução dos níveis para a “zona cinzenta”, ou seja, <600 pg/mL. Em nossa opinião, a paratireoidectomia cirúrgica só é indicada em pacientes nos quais o tratamento médico não tenha sido bem-sucedido^5^.

Pelepenko et al.^7^ mencionam, acertadamente, várias limitações de sua pesquisa. Uma primeira limitação importante é que somente 13% dos centros de diálise no Brasil retornaram o questionário da pesquisa, sendo quase metade deles localizados na região Sudeste do país. Dessa forma, os achados podem não refletir de maneira fiel as regiões do Norte, com status econômico mais baixo. Em segundo lugar, a pesquisa não fornece informações sobre as causas e os fatores envolvidos nas dificuldades relacionadas ao acesso ao tratamento clínico e cirúrgico do HPTS. Terceiro, não há informações sobre o impacto de um controle insatisfatório do HPTS nos desfechos dos pacientes. Em quarto lugar, a disponibilidade insuficiente de cirurgiões de cabeça e pescoço é um tanto surpreendente, já que essa é provavelmente a opção de tratamento mais barata para o HPTS grave. Outras limitações incluem a falta de informações precisas sobre modalidades de terapia de diálise além da hemodiálise e a ausência de uma discussão dedicada aos 18,7% de pacientes com níveis de PTH inadequadamente normais ou baixos (<100 pg/mL) nos quais o risco de mortalidade também aumenta^4^.

Apesar dessas limitações, a pesquisa de Pelepenko et al.^7^ é um documento útil e atual sobre a presente condição do HPTS na DRC e seu manejo no Brasil. Espera-se que ela incite as agências governamentais a tomarem decisões apropriadas na área da saúde, com o objetivo de melhorar ainda mais os desfechos dos pacientes.
